# Does This Look Infected? Hidden Host Plant Infection by the Pathogen *Botrytis cinerea* Alters Interactions between Plants, Aphids and Their Natural Enemies in the Field

**DOI:** 10.3390/insects15050347

**Published:** 2024-05-12

**Authors:** Norhayati Ngah, Rebecca L. Thomas, Mark D. E. Fellowes

**Affiliations:** 1 East Coast Environmental Research Institute, Universiti Sultan Zainal Abidin, Gong Badak Campus, Kuala Nerus 21300, Terengganu, Malaysia; norhayatingah@unisza.edu.my; 2 Faculty of Bioresources and Food Industry, Universiti Sultan Zainal Abidin, Besut Campus, Besut 22200, Terengganu, Malaysia; 3 School of Biological Sciences, University of Reading, Whiteknights, Reading RG6 6AS, UK; 4 School of Biological Sciences, Royal Holloway University of London, Egham, Surrey TW20 0EX, UK

**Keywords:** aphid, parasitoid, predatory insect, plant disease, asymptomatic infection, multitrophic interaction

## Abstract

**Simple Summary:**

Differences in plant quality can alter patterns of ecological interactions, changing complex relationships among organisms within ecological communities. Hidden (asymptomatic) plant disease might have a previously little considered influence on insect ecology. This study investigated how a hidden fungal pathogen affects plants and their associated insects, using two varieties of lettuce as a model system. The presence of hidden disease changed plant quality, affecting insect populations differently depending on the lettuce variety. While the diversity and abundance of aphids remained unaffected in one variety, infection changed aphid assemblages in another. At the same time, aphids on infected lettuce varieties were less attractive to natural enemies, and so this reduced the benefits to aphids of colonising uninfected plants. Understanding the consequences of hidden pathogen infections for plant–insect interactions is important for pest management, biological control and broader ecological science, offering insights into both insect community ecology and sustainable agriculture.

**Abstract:**

Few studies have considered whether hidden (asymptomatic) plant pathogen infection alters ecological interactions at the higher trophic levels, even though such infection still affects plant physiology. We explored this question in two field experiments, where two varieties of lettuce (Little Gem, Tom Thumb) infected with *Botrytis cinerea* were either (1) naturally colonised by aphids or (2) placed in the field with an established aphid colony. We then recorded plant traits and the numbers and species of aphids, their predators, parasitoids and hyperparasitoids. Infection significantly affected plant quality. In the first experiment, symptomatically infected plants had the fewest aphids and natural enemies of aphids. The diversity and abundance of aphids did not differ between asymptomatically infected and uninfected Little Gem plants, but infection affected the aphid assemblage for Tom Thumb plants. Aphids on asymptomatically infected plants were less attractive to predators and parasitoids than those on uninfected plants, while hyperparasitoids were not affected. In the second experiment, when we excluded natural enemies, aphid numbers were lower on asymptomatically and symptomatically infected plants, but when aphid natural enemies were present, this difference was removed, most likely because aphids on uninfected plants attracted more insect natural enemies. This suggests that hidden pathogen infection may have important consequences for multitrophic interactions.

## 1. Introduction

Plant pathogens are ubiquitous in nature and have enormous effects in agriculture, where they cause approximately USD 220 billion of losses to the global economy each year [1]. The most obvious consequence of pathogen infection is the presence of disease symptoms on the plant. For example, necrotrophic pathogens such as *Sclerotinia sclerotiorum* (stem rot) generally kill host cells when they are actively growing, resulting in visible disease symptoms [2]. The presence of symptoms is a critical element in determining how the crop should be managed or the disease should be treated [3]. 

However, the effects of plant pathogen infection extend beyond the expression of symptoms. The presence of pathogens elicits major phenotypic changes in host plant quality [4,5,6], triggering a change in the synthesis or degradation of hormones [7,8,9] and inducing secondary metabolites such as stilbenes and saponins [10,11,12] as the plant defends itself against attack. The expression of this defence mechanism varies with plant genotype and physiology [13]. Some plant varieties are more susceptible to pathogen infection, while some others are resistant [14]. The differences in the strength of plant defence mechanisms amongst plant varieties grown in the same area may influence the severity and timing and also the expression of pathogen attacks, and at the same time such variation will affect interactions between the host plant and species at higher trophic levels. 

As plant traits and quality are key determinants of the growth, development and fecundity of insect herbivores, changes in host plant quality caused by the presence of a plant pathogen can in turn have consequences for the growth, fitness and behaviour of insect herbivores [15]. Chemical defences produced by the plants during pathogen attack (such as anti-nutritive or toxins) may suppress the growth and influence the behaviour of herbivores [16]. Plant volatiles produced by the infected plant may influence how insects locate resources required for nutrition and reproduction, as many insects rely on olfactory and visual cues [17,18]. These changes may have either a positive or negative effect, depending on the characteristics of the insect species concerned [19]. 

In turn, such changes in plant–herbivore interactions can also affect the behaviour and abundance of the insect herbivores’ natural enemies [16,20]. Taken together, it is therefore not surprising that overt plant pathogen infection can alter the structure of insect communities [21]. However, not all plant pathogen infections result in visible symptoms, and there is an absence of studies which examine in detail the consequences of asymptomatic infections on how ecological networks form and the subsequent patterns seen in ecological interactions at higher trophic levels.

*Botrytis cinerea* Persoon: Fries s. lato is a ubiquitous necrotrophic plant pathogen responsible for grey mould disease, impacting over 1000 hosts worldwide [22]. It affects photosynthesis, reduces plant weight and triggers the production of secondary metabolic defence compounds in host plants [23]. Infection can induce the activation of jasmonic acid (JA)- and ethylene (ET)-dependent defence signalling [24], with salicylic acid (SA)-induced pathways only becoming important later if the necrotroph starts to behave as a hemi-biotroph [25]. This pathogen’s effects extend beyond plant health, influencing plant–insect interactions by altering nutrient values and inducing defence reactions in plants, indirectly impacting the performance and behaviour of insect herbivores [26]. Such interactions involve complex cross-talk, potentially altering outcomes [27,28]. For instance, *Botrytis* infection inhibits the development, survival and fecundity of *Aphis fabae* (Homoptera: Aphididae), with effects intensifying with lesion density [26]. However, *B. cinerea* infection may also enhance aphid performance, as infected host plants offer an enriched diet [29,30,31]. These varying aphid responses stem from differences in nitrogen content in *Botrytis*-infected leaves and the availability of amino acids resulting from pathogen-induced senescence in phloem mesophyll cells [26].

However, pathogen infection does not always result in visible symptoms. While *B. cinerea* causes considerable economic losses through visible damage, this pathogen can also exist as an asymptomatic infection in the host plant [32]. Plants are identified as having asymptomatic *Botrytis* infection when there are no visible symptoms of pathogen infection such as soft rots or lesions, but infection is confirmed to be present when assayed using *Botrytis*-selective medium (BSM) agar. Current findings show that frequent systemic and asymptomatic infection by *B. cinerea* is widespread and has been observed in cultivated primula and cyclamen [33,34], lettuce [35] and soft fruit such as strawberries [36,37] and grapes [38,39,40]. This may be due to variations in host plant susceptibility to *Botrytis* infection [41]. Furthermore, different strains of *B. cinerea* exhibit varying degrees of virulence, affecting their ability to establish latent infections within plant tissues [42]. In addition, environmental factors such as temperature, light level, humidity and nutrient availability play a crucial role in fungal growth and colonization within plant tissues [43,44]. It has previously been reported that endophytic interactions and induced resistance responses further influence the establishment of latent infections [45]. The mechanism of asymptomatic infection by this pathogen on lettuce is now understood [35], but we have little understanding of the wider ecological consequences of the presence of hidden, asymptomatic infection. 

Our previous work in the laboratory showed that asymptomatic infection by *B. cinerea* in lettuce causes slight, albeit measurable, changes in host plants, and this varies between plant varieties. Aphids (*Myzus persicae* Sulzer; Hemiptera: Aphididae) reared on asymptomatically infected lettuce plants were smaller, had fewer offspring and were less tolerant of starvation; parasitoids (*Aphidius eadyii* Viereck; Hymenoptera: Braconidae) emerging from such hosts were also smaller [15]. When given a choice in an olfactometer, both aphids and parasitoids preferentially chose uninfected plants, and aphids were more likely to show escape (dropping) behaviours in response to foraging coccinellid predators when reared on asymptomatically infected plants [15]. Together, these results suggest that hidden *B. cinerea* infection could greatly influence patterns of species interactions in the field.

To address this, we examined the consequences of asymptomatic and symptomatic pathogen infection for the structure of naturally formed insect communities at higher trophic levels in two field experiments, both using two varieties of the host plant. First, in our sentinel experiment, we asked if host plant infection affected the recruitment of aphids and their natural enemies, where insects were allowed to colonize uninfected and infected plants naturally in the field. Second, to further explore the effects of pathogen infection on aphid–natural enemy interactions, we placed experimental plants in the field with established aphid (*Myzus persicae*) colonies. In both trials, we recorded (i) plant traits; (ii) aphid diversity and abundance; and (iii) the abundance and diversity of aphid natural enemies found on (a) control (uninfected); (b) infected but symptom-free and (c) infected and symptomatic plants (our established colony experiment). 

## 2. Materials and Methods

### 2.1. Study System

Plants and infection status. Two lettuce *Lactuca sativa* L. (Asteraceae) varieties (Tom Thumb and Little Gem) were used. The varieties differ in their morphology and leaf characteristics. Tom Thumb has smoother leaves with a more compact arrangement than Little Gem. Little Gem is larger and wider than Tom Thumb and grows to about 10 cm wide and 15 cm tall. These two varieties were used as host plants as they are susceptible to asymptomatic *B. cinerea* infection [35]. In experiments in controlled environments, these varieties were also equally attractive to aphids [15]. Plants were grown from pathogen-free and *B. cinerea*-infected seeds (following [35]). Infected seeds were collected from plants which were systemically infected with the *B. cinerea* strain BO5.10 spores during their flowering stage, while uninfected seeds were collected from uninfected plants. Both uninfected and *B. cinerea*-infected plants for seed collection were grown in 2014 in different glasshouses under the same conditions to avoid pathogen cross-contamination. 

Experimental plants were grown from seeds sown in individual cells of plug trays with professional seed and modular compost (Clover brand; Dungannon, UK) in a glasshouse (temperature: 25–30 °C; relative humidity: 80 ± 5%; and L12:D12 photoperiod). Fourteen days after emergence, seedlings were transferred into 15 cm diameter pots with traditional potting compost (Vitax Grower; Leicester, UK). Sixty replicates were grown per treatment in each experiment, as infection in the *Botrytis*-treated seeds and lack of infection in the control plants was not guaranteed. Plants were then allowed to grow for another four weeks in the glasshouse and go through a hardening process for three days under shade before planting in the experimental grounds. 

Plants that showed symptoms of pathogen infection were discarded immediately to avoid pathogen contamination in the glasshouse. Two weeks before each experiment started, the infection status of the experimental plants was checked using *Botrytis*-selective medium (BSM) agar. Thirty *Botrytis*-infected/uninfected plants were then selected randomly from the tested plants for use in each experiment. Six-week-old plants free from any symptoms of infection were used in this study. It should be noted that some plants were lost due to attacks by slugs and deer during the experiment.

Plants were treated equally and plants which subsequently showed symptoms of pathogen infection were allowed to continue developing naturally during the course of the experiment. Prior to harvest at the end of the experiments, the pathogen infection status of the plants (either symptomatic or asymptomatic) was recorded. We categorised “symptomatic plants” as plants that showed either restricted or dry lesions or spreading soft rots with or without the appearance of conspicuous sporulating colonies [46], while “asymptomatic plants” were categorized as plants grown from infected seeds but not showing any of the above symptoms, and the infection status of all plants was tested as described below at the end of the field trials. 

Aphids. Our model herbivore was a single clone of the green peach aphid *Myzus persicae* Sulzer (Hemiptera: Aphididae). This is a generalist phloem feeder, which had previously been reared for four generations on *Botrytis*-infected or uninfected plants prior to use to avoid confounding maternal effects. Aphids were reared in the laboratory at ambient temperature, isolated using cylindrical clear plastic cages fitted with cotton mesh windows. Each experimental aphid colony was established with five seven-day-old aphids which were placed on the plants five days before being transferred to the field site.

Field site. The experiments were conducted at the experimental grounds, University of Reading, United Kingdom (51.4414° N, 0.9418° W). The site was surrounded by crop and ornamental plants (e.g., broad bean and strawberry) with naturally occurring populations of aphids, as well as nearby gardens, glasshouses and the university buildings. Plants were randomly placed in a grid pattern in a field site within the experimental grounds and arranged approximately 1.7 m apart to reduce interactions between plants undergoing different treatments. Plants were watered as required and weeds were removed. Each plant pot was placed in a shallow plastic dish to minimize the effect of water stress or saturation. 

### 2.2. Sentinel Experiment

Experiments were carried out twice (May and July 2016) to capture variation in aphid abundance. Each treatment (two lettuce varieties; infected or uninfected) was replicated 30 times, with 120 plants per experiment (240 in total). The numbers of aphids, parasitoid mummies and predatory insects present on each plant were recorded every two days until all aphid colonies died, which took approximately 28 days. The mummies, predatory insects and a sample of aphids observed from each species were collected and identified in the laboratory. If more than one mummy was found on the plant, half (randomly chosen) were left for up to 72 h on the plant to allow for hyperparasitoid attack. 

### 2.3. Established Aphid Colony Experiment

The experiments were carried out in May 2015. There were three treatments (plant variety, infection status, and exposure to natural enemy attack). Each treatment was replicated 30 times, with 240 plants and aphid colonies initially placed in the study site. Non-predation plants were covered with breathable plastic bags to protect aphids from natural enemies, allowing the effects of plant infection status on aphid colony growth to be assessed. Data were recorded as described above. 

### 2.4. Plant Traits

Plant traits were measured at the end of each experiment. Chlorophyll content was measured on three randomly chosen leaves of each replicate plant, using a handheld Chlorophyll Meter (Model atLeaf; FT Green LLC, Wilmington, DE, USA) before the plant was harvested. The height of the plants was measured on the first day and on the final day of each experiment. The plants (including roots) were harvested and dried in an oven at 75 °C until reaching constant mass (~48 h), and they were then weighed using an electronic balance (Sartorius, LC 6200S, Goettingen, Germany). The root/shoot ratio was calculated by dividing the dry weight of shoots by the root dry weight for each plant.

### 2.5. Assessing B. cinerea Infection

Following data collection, plants were visually inspected for symptoms of disease (following [46]). All plants (both experimentally infected and uninfected prior to placement) were then assessed for the presence of *B. cinerea*. Three 1 cm diameter mature leaf samples with no visible symptoms of infection were randomly harvested at the end of the experiment from each plant. The leaf samples were sterilised before plating on *Botrytis*-selective medium (BSM) agar to confirm the *Botrytis* infection status of the plants [47]. Leaf samples were disinfected with 70% ethanol for 1 min, followed by 1 min in 2% bleach solution (Domestos, Unilever; 5% NaOCl in alkaline solution with surfactants) and then rinsed three times in sterile distilled water to remove all of the surface inoculum [34]. The sterile leaf disk then was plated on BSM agar and incubated at 18–20 °C in an incubator with alternating UV-A light (12:12 h light/dark). After fourteen days, the BSM plate was observed again to see whether there was evidence of *B. cinerea* growth. Confirmation of presence was based on the sporulation of the pathogen and morphological observation of the colonies under a high-performance stereomicroscope (Leica, MZ9.5, TX, USA). Plant health status was therefore categorized as (i) symptomatic infection if symptoms of infection were visible and presence was confirmed by the BSM agar test; (ii) asymptomatic infection if there were no visible symptoms of *Botrytis* infection, but the plated BSM agar showed *Botrytis* growth; and (iii) healthy if no symptoms of *Botrytis* infection appeared on the plant and there was no sign of fungal growth on the BSM agar.

### 2.6. Statistical Analyses

All data were analysed using R statistical software version 3.4.0 [48]. Linear mixed effects models with a restricted maximum likelihood method were calculated to investigate the influence of plant variety and plant pathogen infection on plant traits (chlorophyll content, dry weight, plant height and shoot/root ratio).

Sentinel experiment. The count data for aphids, parasitoid mummies and predators were pooled across time and the cumulative number of aphids was analysed using generalized linear mixed models using the glmmADMB package [49] and negative binomial family as the data were over-dispersed [50]. The effects of plant variety, infection status and the cumulative number of aphids on the numbers of predators and parasitoid mummies collected were analysed using glmer.nb with a Poisson distribution [51], where aphid number was treated as a covariate. The action of secondary parasitoids on mummies found on the plant was also investigated by using similar analysis with the number of mummies treated as a covariate. In all analyses, the time of experiment was treated as a random effect. The significance of differences between mean values was determined by using LSmeans and separation by post hoc Tukey tests using the plant variety and infection status as explanatory variables. Species diversity was estimated according to the Shannon diversity index using the vegan package [52]. 

The structure of ecological networks was analysed using the econullnetr package [53]. This R package is a null model approach that estimates interaction strengths for each pair of resource and consumer species in a network based on the modelled resource selection for each individual consumer. Four matrix parameters to measure the ecological networks were calculated in bipartite networks (nestedness, linkage density, connectance and interaction evenness). The analyses on the structure of ecological networks and the selectivity test for aphid, predator and parasitoid species towards plant treatments were performed separately for each plant variety. 

Established aphid colony experiment. The effect of plant variety, natural enemy exposure and pathogen infection on the number of aphids was analysed using a repeated measures analysis with generalized least square’s function. The effects of plant variety and pathogen infection on the numbers of predators and parasitoid mummies collected were analysed using generalized linear models with a Poisson distribution, where aphid number was treated as a covariate. Differences between mean values of plant traits and the number of aphids were examined using LSmeans and separation by post hoc Tukey test, with plant variety, infection status and plant exposure to aphid natural enemies treated as explanatory variables. Analysis of the structure of ecological networks and the selectivity test for the aphid colony experiment were performed as described for the sentinel experiment. 

## 3. Results

### 3.1. Sentinel Experiment

Plant traits. The lettuce varieties differed in their physical traits (Table 1). Overall, infection by *B. cinerea* (either symptomatic or asymptomatic) substantially reduced the chlorophyll content, plant height and shoot/root ratio of Little Gem plants, but there were no significant differences in plant traits for uninfected and asymptomatic Tom Thumb plants (Table 2). Symptomatic Tom Thumb plants were found to have a lower chlorophyll content and were smaller than uninfected Tom Thumb plants (Table 2).

Aphid numbers. The cumulative number of aphids counted was 26,427 individuals, consisting of *Myzus persicae* (Sulzer), *Myzus ornatus* (Laing)*, Macrosiphum euphorbiae* (Thomas)*, Acyrthosiphon lactucae* (Passerini) and *Aphis fabae* (Scopoli) (Table 3). Aphid diversity (Shannon H) was highest on uninfected Tom Thumb plant (1.24), followed by infected Little Gem (1.09), infected Tom Thumb (1.00) and uninfected Little Gem plants (0.94).

**Figure 1:**
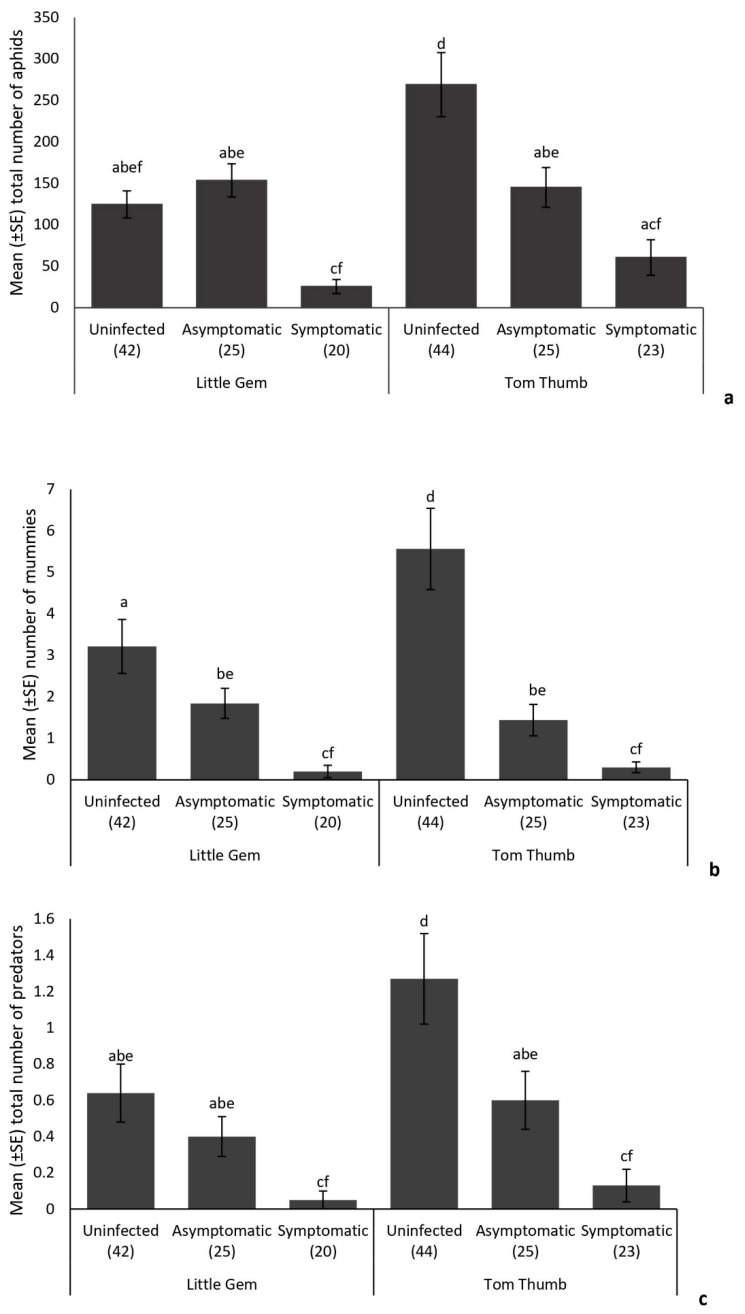
Influence of the lettuce infection status (uninfected/symptomatic/asymptomatic) and variety on the mean (+/−SE) number of (**a**) aphids, (**b**) parasitoid mummies and (**c**) predators found on plants in the sentinel experiment. Treatments sharing the same letters above each bar are not significantly different at *p* < 0.05 following post hoc tests.

There was no significant difference in overall aphid abundance between plant varieties (Table 4). The number of aphids recorded on infected (both symptomatic and asymptomatic) Tom Thumb plants was lower than that found on uninfected Tom Thumb plants (Figure 1). Asymptomatic infection of *B. cinerea* in Little Gem plants did not significantly affect the number of aphids when compared with those found on uninfected plants (Figure 1), but aphid abundance was significantly lower on plants showing symptoms of *Botrytis* infection. 

**Figure 2:**
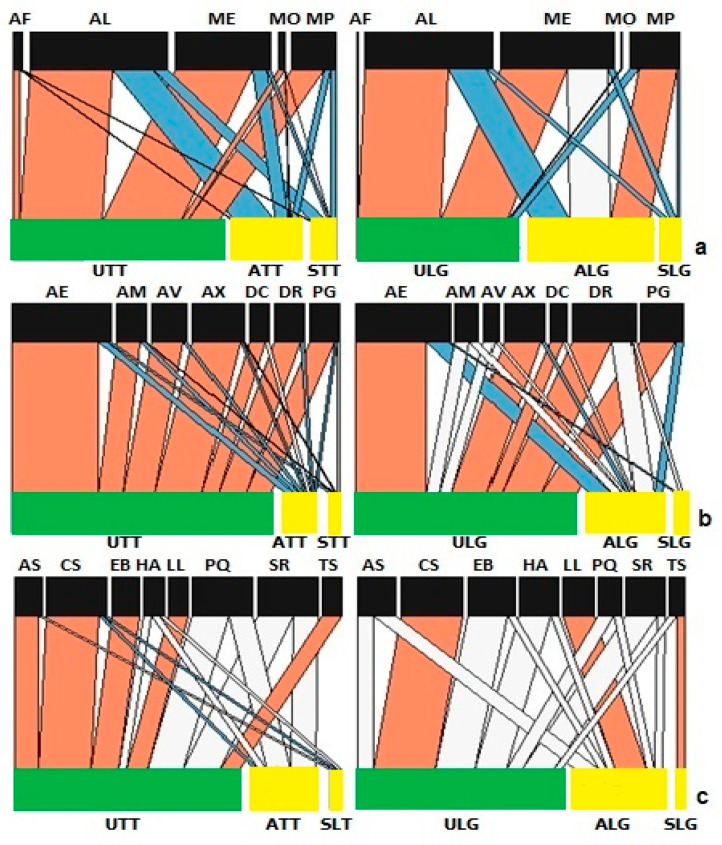
Network analysis of species found on lettuce in the sentinel experiment. Link widths represent the observed frequency of interactions, with red links being stronger and blue links being weaker than expected compared to the null model. The white links represent no significant differences. Bar widths at the two levels indicate the relative abundances of species at different trophic levels for lettuce and (**a**) aphids (AF, *Aphis fabae*; AL, *Acyrthosiphon lactucae*; ME, *Macrosiphum euphorbiae*; MO, *Myzus ornatus*; MP, *Myzus persicae*); (**b**) parasitoids (AE, *Aphidius ervi*; AM, *Aphidius matricariae*; AV, *Asaphes vulgaris*; AX, *Alloxysta victrix*; DC, *Dendrocerus carpenteri*; DR, *Diaeretiella rapae*; PG, *Praon gallicum*); and (**c**) predators (AS, *Anthocoris nemorum*; CS, *Coccinella septempunctata*; EB, *Episyrphus balteatus*; HA, *Harmonia axyridis*; LL, lacewing; PQ, *Propylea quatuordecimpunctata*; SR, *Syrphus ribesii*; TS, *Tachyporus* sp.). Abbreviations—U: uninfected plants; A: asymptomatic infected plants; S: symptomatic infected plants; LG: Little Gem variety; TT: Tom Thumb variety.

Analysis of the structure of aphid networks for aphid species on Tom Thumb plants showed significant differences in nestedness, linkage density, connectance and interaction evenness (Supplementary Table S1), but patterns differed from those found on Little Gem plants (Supplementary Table S2). Uninfected Tom Thumb plants displayed a strong interaction with all species of aphids, while the *Botrytis-*infected Tom Thumb plants, either symptomatic or asymptomatic, did not (Figure 2). Notably, *Myzus persicae* is the only aphid species that had a strong interaction with asymptomatic Little Gem plants, but not with uninfected plants. 

#### Natural Enemies

Parasitoids: A total of 473 mummies were collected in these experiments. Overall, 414 of these mummies emerged; four species were identified as primary parasitoids (53.72%) and three as secondary parasitoids (46.28%) (Table 3). The primary parasitoids were all members of the Family Braconidae [*Aphidius ervi* (Haliday), *Aphidius matricariae* (Haliday), *Praon gallicum* (Stary), *Diaeretiella rapae* (M’Intosh), while the hyperparasitoids were *Dendrocerus carpenteri* (Curtis) (Hymenoptera: Ceraphronidae), *Asaphes vulgaris* (Walker) (Hymenoptera: Pteromalidae) and *Alloxysta victrix* (Westwood) (Hymenoptera: Cynipidae). The polyphagous parasitoid *A. ervi* was the most abundant species, with 136 individuals (32.85% of all individuals), followed by the secondary parasitoid, *A. victrix* (15.94%) (Table 3). Parasitoid diversity (Shannon H) was highest on infected Tom Thumb plants (1.81), followed by uninfected Tom Thumb (1.79), uninfected Little Gem (1.75) and infected Little Gem plants (1.66). 

While plant variety did not influence the number of mummies, the number of aphids and plant pathogen infections affected the number of mummies recorded (Table 4). Infection by *B. cinerea* was associated with a reduction in the number of mummies on both plant varieties. The number of mummies was also reduced on asymptomatic plants of both varieties (Figure 1). The numbers of hyperparasitoids were not influenced by plant variety or pathogen infection, but were affected by the numbers of mummies present. Analysis of the structure of parasitoid networks for both Tom Thumb and Little Gem plants showed significant differences in nestedness, linkage density, connectance and interaction evenness, associated with infection status (Supplementary Tables S1 and S2; Figure 2). 

Predators: A total of 112 predatory insects (larvae and adults) were collected over both experimental periods (Table 3). These insects include the coccinellid ladybirds *Harmonia axyridis* (Pallas)*, Propylea quatuordecimpunctata* (Linnaeus) and *Coccinella septempunctata* (Linnaeus); the hoverflies *Episyrphus balteatus* (De Geer) and *Syrphus ribesii* (Linnaeus); flower bugs *Anthocoris nemorum* (Linnaeus); rove beetles *Tachyporus* sp; and lacewings (Family: Chrysopidae). Ladybirds were the most abundant predatory insect collected in the experiment (45.53% of all individuals). All species of ladybirds were found on most of the plant types, except for *C. septempunctata*, which were absent from infected Little Gem plants. The abundance of hoverfly larvae was also high (29.46%), followed by flower bugs (10.71%), lacewings (8.03%) and rove beetles (6.25%) (Table 3). There were no lacewings or rove beetles found on infected Tom Thumb plants. The diversity of predatory insects (Shannon H) was highest on uninfected Tom Thumb plants (1.97), followed by uninfected Little Gem (1.77), infected Little Gem (1.74) and infected Tom Thumb plants (1.43). 

The number of predatory insects was not influenced by the number of aphids but was affected by plant variety (Table 4), with fewer predators collected on Little Gem plants (Figure 1). Overall, more predatory insects were recorded on uninfected plants than on infected plants, and there were significantly more on uninfected than on asymptomatic plants (Figure 1). Taken together, plant variety (Tom Thumb, Little Gem) and plant infection status (uninfected, infected but asymptomatic and infected and symptomatic) influenced the natural assemblages of insect herbivores and their natural enemies found on the experimental plants (Figure 1 and Figure 2).

Analysis of the structure of predator networks on Tom Thumb plants showed that nestedness was significantly higher while linkage density, connectance and the interaction evenness were significantly lower than expected (Supplementary Table S1). Uninfected Tom Thumb plants showed strong interactions with most species of predators (Figure 2). On the other hand, asymptomatic Tom Thumb plants showed weaker interactions with *A. nemorum, C. septempunctata, E. balteatus* and *Tachyporus* sp., while symptomatic Tom Thumb plants showed weaker interactions with two species of ladybirds (*C. Septempunctata and P. quatuordecimpunctata*) and the hoverfly *S. ribesii* (Figure 2). In contrast, Little Gem plants showed no significant difference in nestedness, but linkage density, connectance and the interaction evenness were significantly lower than expected (Supplementary Table S2). Most predators displayed no significant effect from plant infection status, with the exceptions of the ladybird *C. septempunctata*, lacewings and *Tachyporus* sp. (Figure 2). 

### 3.2. Established Aphid Colony Experiment

*Plant traits.* Lettuce varieties differed in chlorophyll content, dry weight, plant height and shoot/root ratio (Table 5). *Botrytis cinerea* infection, whether symptomatic or asymptomatic, reduced the chlorophyll content of lettuce plants (Table 6). Both symptomatic and asymptomatic pathogen infection only affected the dry weight of protected Little Gem plants, and did not show any significant effect on Tom Thumb plants (Table 6). On the other hand, symptomatic and asymptomatic pathogen infection affected the height of protected Tom Thumb plants and did not influence the height of Little Gem plants (Table 6). The effect of pathogen infection on plant dry weight (Little Gem) and plant height (Tom Thumb) was eliminated when the plant was exposed to aphid natural enemies. If protected, the shoot/root ratio of uninfected Little Gem plants was significantly higher than asymptomatic or symptomatic Little Gem plants. These results were the inverse when Little Gem plants were exposed to aphid natural enemies, where the shoot/root ratio of exposed uninfected Little Gem plants is lower than that of symptomatic and asymptomatically infected Little Gem. 

**Figure 3:**
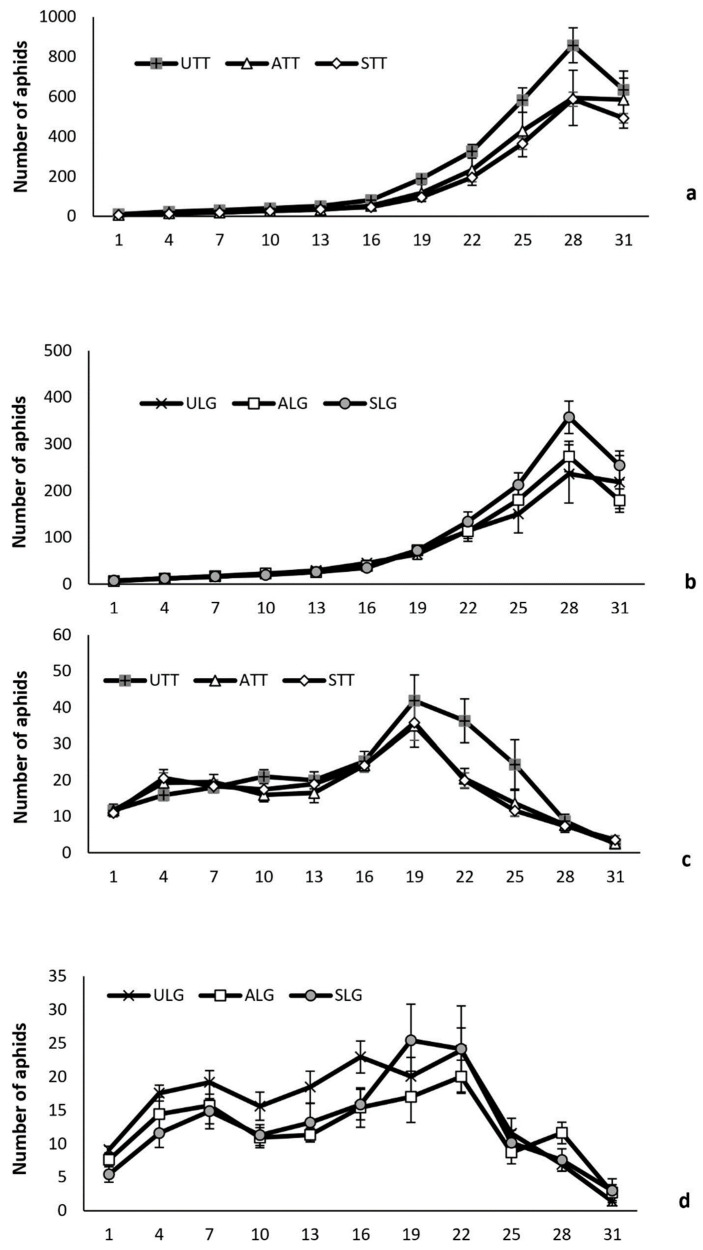
Influence of the infection status (uninfected/symptomatic/asymptomatic) and plant variety on the mean (+/−SE) number of aphids on lettuce plants per recording day in the established aphid colony experiment. Aphids recorded on plants where protected from (**a**,**b**) or exposed to aphid natural enemies (**c**,**d**). Abbreviations—U: uninfected plants; A: asymptomatic infected plants; S: symptomatic infected plants; LG: Little Gem variety; TT: Tom Thumb variety.

*Myzus persicae* abundance. Overall, plant pathogen infection, plant variety and natural enemy attack influenced the number of aphids of plants (Table 7). More aphids were recorded on Tom Thumb plants than on Little Gem plants for both exposed and protected plants (Figure 3). Pathogen infection, either symptomatic or asymptomatic, reduced aphid numbers, and this effect changed over time and differed between plant varieties (Figure 3). When protected from attack by natural enemies, the number of aphids on uninfected Tom Thumb plants was significantly higher than on asymptomatic and symptomatic Tom Thumb plants. However, the number of aphids on protected uninfected, asymptomatic and symptomatic Little Gem plants showed no significant difference. The number of aphids on all plant treatments was greatly reduced when exposed to the attack by their natural enemies (Figure 3). No significant differences were detected for the numbers of aphids on uninfected, symptomatic and asymptomatic plants for both lettuce varieties. 

#### Natural Enemies

**Figure 4:**
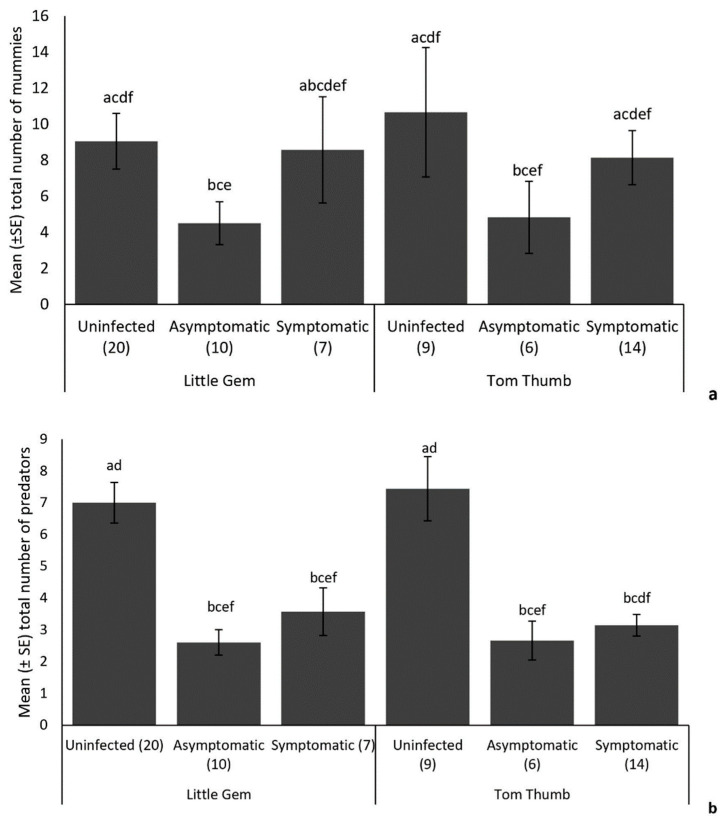
Influence of the lettuce infection status (uninfected/symptomatic/asymptomatic) and variety on the mean (+/−SE) number of (**a**) parasitoid mummies and (**b**) predators found on plants in the established aphid colony experiment. Treatments sharing the same letters above each bar are not significantly different at *p* < 0.05 following post hoc tests.

Parasitoids. We collected 525 parasitoid mummies, among which 394 emerged and were identified as belonging to five species (the braconids *Aphidius ervi* Haliday, *Aphidius matricariae* Haliday, *Praon gallicum* Stary, *Diaeretiella rapae* M’Intosh and the pteromalid *Asaphes vulgaris* Walker) (Table 8). *Aphidius ervi* was the most abundant (27.66%) species, followed by *A. matricariae* (25.89%), *P. gallicum* (25.63%) and *D. rapae* (12.44%). The hyperparasitoid *As. vulgaris* comprised 8.38% of records. The number of parasitoid mummies recorded on plants was significantly affected by *B. cinerea* infection, plant variety and the number of aphids on the plant (Table 9). More parasitoid mummies were recorded on uninfected and symptomatic plants compared to asymptomatic plants for both lettuce varieties (Figure 4). 

**Figure 5:**
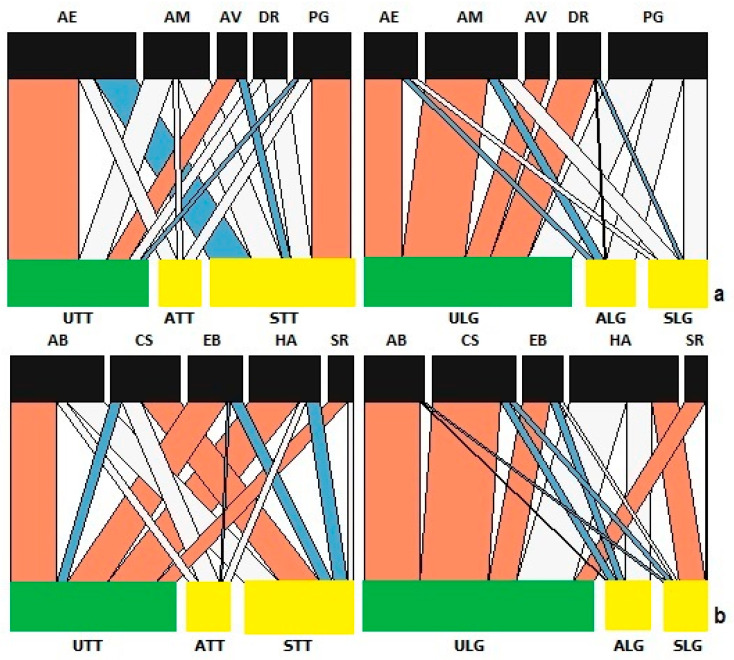
Network analysis of species found on lettuce in the established aphid colony experiment. Link widths represent the observed frequency of interactions, with red links being stronger and blue links being weaker than expected compared to the null model. The white links represent no significant differences. Bar widths at the two levels indicate the relative abundances of species at different trophic levels for lettuce and (**a**) parasitoids (AE: *Aphidius ervi*; AM: *Aphidius matricariae*; AV: *Asaphes vulgaris*; DR: *Diaeretiella rapae*; PG: *Praon gallicum*) and (**b**) predators (AB: *Adalia bipunctata*: CS: *Coccinella septempunctata*; EB: *Episyrphus balteatus*; HA: *Harmonia axyridis*; SR: *Syrphus ribesii*). Abbreviations—U: uninfected plants; A: asymptomatic infected plants; S: symptomatic infected plants; LG: Little Gem variety; TT: Tom Thumb variety.

The structure of the assemblage of parasitoid species differed with host plant infection status in nestedness, linkage density and connectance, but not interaction evenness (Supplementary Tables S3 and S4). The effect of infection status on parasitoids differed considerably between host plants, driven largely by *Praon gallicum* and *Aphidius ervi* (Figure 5). 

Predators. A total of 315 predators were observed in this experiment, mainly consisting of predatory ladybirds (79.05%) and hoverfly larvae (20.95%). The ladybirds were *Harmonia axyridis* Pallas, *Coccinella septempunctata* Linnaeus and *Adalia bipunctata* Linnaeus, while hoverfly larvae were *Episyrphus balteatus* De Geer and *Syrphus ribesii* Linnaeus (Table 8). *Harmonia axyridis* was the most abundant predator (30.16%), followed by *C. septempunctata* (25.08%), *A. bipunctata* (23.81%), *E. balteatus* (14.60%) and *S. ribesii* (6.35%). Overall, there was no significant difference in the number of predators between plant varieties (Table 9). Aphid numbers did not influence the number of predatory insects collected. However, symptomatic and asymptomatic infection by *B. cinerea* did affect the number of predators observed on both plant varieties, with significantly more predators recorded on uninfected plants (Figure 4). 

Analysis of the networks of predator species showed that plant variety and infection status affected linkage density, connectance and interaction evenness, and the nestedness for predators on Tom Thumb plants is not significantly different, but the nestedness statistic for Little Gem plants is significantly higher (Supplementary Tables S3 and S4). Notably, *H. axyridis* is more likely to be found on symptomatic Little Gem plants but is less likely to be found on symptomatic Tom Thumb plants, while *C. septempunctata* showed strong interactions with symptomatic Tom Thumb plants but not symptomatic Little Gem plants (Figure 5). 

## 4. Discussion

There is little doubt that symptomatic plant pathogen infection alters interactions between host plants and their herbivores. Here, we show that asymptomatic infection with the ubiquitous and economically important plant pathogen *B. cinerea* can affect interactions between its host plant, the plant’s herbivores and the natural enemies of these herbivores in the field. This suggests that even when no symptoms of host infection are present, plant pathogens can have substantial effects on the structure of terrestrial communities, and this may also have implications for approaches to crop protection. 

Plant quality was affected by *Botrytis* infection status and plant variety. In both experiments, Little Gem plants had a greater chlorophyll content, height and dry mass than Tom Thumb plants. In the sentinel experiment, we found that asymptomatic *B. cinerea* infection reduces the chlorophyll content (likely a result of lost nitrogen levels [54]), plant height and shoot/root ratio of Little Gem plants, but found no difference in dry weight. No effect of asymptomatic *Botrytis* infection was detected in Tom Thumb plant traits. In the established aphid colony experiment, the protection of aphids from attack by their natural enemies indirectly influenced the traits of both plant varieties. These changes might be influenced by the interaction between the numbers of aphids and the effects of pathogen infection on the protected plant. Asymptomatic infection by *B. cinerea* in protected plants changed the chlorophyll content, plant dry weight and shoot/root ratio of Little Gem plants, and only affected the chlorophyll content and height of Tom Thumb plants. On the other hand, when exposed to aphid natural enemies, asymptomatic *B. cinerea* infection reduced the chlorophyll content of Little Gem and Tom Thumb plants. This suggests that the effects of asymptomatic infection are subtle, and when environmental variation is present, they may be masked. 

In turn, pathogen infection affected the numbers and diversity of insects recorded on the study plants. In the sentinel experiment, both symptomatic and asymptomatic pathogen infection on Tom Thumb reduced the number of aphids colonizing the plants, and in turn indirectly influenced the number of natural enemies. However, with Little Gem plants, the presence of asymptomatic pathogen infection did not influence the number of aphids, but did affect the number of natural enemies, while symptomatically infected Little Gem plants harboured fewer aphids, which affected the numbers and diversity of predators and parasitoids. 

In the established colony experiment, when we prevented natural enemy attack, we found that both symptomatic and asymptomatic pathogen infection affected the number of aphids, but this effect varied with plant variety. However, this effect of pathogen presence was eliminated when aphids were exposed to natural enemies. Here, more parasitoid mummies and predators were recorded on uninfected plants. This finding suggests that uninfected plants received more natural enemies compared to symptomatic and asymptomatic plants, thus reducing the numbers of aphids. This points to a complex interplay between host plant traits, infection status and aphid physiology and behaviour, which then influences the recruitment of predators and parasitoids in the field. 

In the sentinel experiment, both varieties of plants attracted five species of aphids. All aphid species were equally attracted to both uninfected and infected Tom Thumb plants. However, *A. fabae* and *M. ornatus* were not observed on infected Little Gem plants. The discrimination of these aphid species against *Botrytis*-infected plants may be related to plant nutrient quality or to a repellent effect resulting from pathogen infection. Aphids show a strong response to nitrogen levels in their host plants [54,55], and in the laboratory, asymptomatic *B. cinerea* infection reduced the size, fecundity and longevity of the aphid *M. persicae*, while parasitoids (*Aphidius colemani*) reared on these aphid hosts showed reduced rates of mummy formation and their offspring were smaller and had reduced starvation resistance [15]. This suggests that these differences in the field are driven by reduced plant quality.

Insect diversity and abundance are influenced by resource quality, competition and the action of natural enemies [56,57]. Here, the decrease in natural enemy abundance on the lettuce variety Tom Thumb is associated with the *Botrytis* infection and the availability of aphids on the plants. Pathogen infection did not affect the number of aphids on Little Gem plants, but did reduce the number of natural enemies. This could be because the pathogen infection was lower, reducing the quality of the aphids, and as a consequence, they attracted fewer natural enemies [58]. 

Plant traits play an important role in determining the dynamics and structure of insect communities. Changes in plant traits induced by the infection of a plant pathogen may have a cascading effect on both the direct and indirect interactions between the plant and other organisms at higher trophic levels. As a consequence, these interactions could shape community structure and influence the abundance of other species within this ecosystem. 

Our study shows that under field conditions, plant pathogen infection may alter the assemblage of insects found on host plants. The strength and consequences of infection depends on the presence of symptoms and the genotype of the infected plant, but critically, the effects of infection can be present even when there are no overt symptoms of disease. While it is still unclear how this system works, the results obtained suggest that systemic, asymptomatic and seed-borne infection by *B. cinerea* can influence the abundance and diversity of aphids and their predators and parasitoids in the field. 

Changes in aphid performance may have a consequential effect on the life history and behaviour of predators and parasitoids [59,60]. Parasitoid oviposition preference is affected by fitness costs in terms of opportunity time, energy, mortality risk and potential fitness returns from oviposition in a particular host [61,62,63]. Similarly, female insect predators also optimise fitness by choosing oviposition sites and the availability of prey that contribute more to lifetime fitness for their offspring [64,65,66]. Predator preference behaviour is affected by aphid-associated chemical stimuli, aphid colony size, the spatial position of the aphid colony and host-plant characteristics [67,68,69,70].

In our experiments, we found that the number of both parasitoid mummies and predatory insects was higher on uninfected plants than on asymptomatically *Botrytis*-infected plants, despite there being no significant difference in aphid numbers. Unexpectedly, even though parasitoids parasitized more aphids on uninfected plants, we found no difference in the numbers of mummies recorded on uninfected and symptomatic plants. On the other hand, symptomatic and asymptomatic plants showed a similar effect in reducing the numbers of predatory insects compared to uninfected plants. While previous studies have found that symptomatic plant pathogen infection affects natural enemy foraging behaviour [71], our findings suggest that variation in plant quality resulting from either symptomatic or asymptomatic infection by *B. cinerea* influences the foraging behaviour of insect natural enemies [72,73,74,75]. 

It is possible that this influence on the foraging behaviour of insect natural enemies does not result from the presence of the pathogen alone, and instead is a result of the co-occurrence of both the pathogen and herbivorous insects [16,75]. Infection by *B. cinerea* triggers the activation of jasmonic acid (JA)- and ethylene (ET)-dependent defence signalling in the plant body [76], while aphid infestation triggers the production of salicylic acid (SA)-dependent pathways [77]. Both pathways do not exist in isolation and cross-talk between these pathways may occur, with activation of the SA-dependent pathway leading to a down-regulation of the JA-dependent pathway and vice versa [4]. 

If so, a change in the emission of many compounds should be the result, and members of the third trophic level may adapt their responses to optimize exploitation of the signals. Since the parasitoid olfactory system is dependent on the chemical pathways produced by the host or host habitat [62], the down-regulation of defence pathways may affect parasitoid foraging behaviour. In our study, we did not measure the quality and quantity of defences metabolites produced by the plant, but it is likely that symptomatic and asymptomatic infection of *B. cinerea* influences plant secondary defences, and this in turn influences the structure of the assemblage of the aphid natural enemies in this system.

## 5. Conclusions

In nature, plants are almost constantly exposed to attack by herbivorous insects and pathogenic microorganisms. While attack by external herbivores can readily be recorded, this is not necessarily so for asymptomatic infection by plant pathogens. As infection by hidden plant pathogens has the potential to shape the composition of insect communities across the landscape and can generate changes which have ramifications at higher trophic levels, this study provides novel insights to help improve our understanding of how these complex systems work. The findings from this experiment contribute to our understanding of the complex network of direct and indirect interactions between plants, pathogens, herbivorous insects and their natural enemies. 

## Figures and Tables

**Table 1:** Summary of the effects of lettuce variety and *B. cinerea* infection status on plant traits for the sentinel experiment. Significant values are in bold.

Plant Traits	Explanatory Variable	Coefficient t Value ± SE	*p*
atLEAF value	**Intercept**	**8.026 ± 3.569**	**<0.001**
	**Variety**	**−3.040 ± 1.506**	**0.002**
	**Uninfected**	**4.946 ± 1.329**	**<0.001**
	Symptomatic	−1.579 ± 1.596	0.116
	**Variety-Uninfected**	**−2.785 ± 1.886**	**0.006**
	Variety-Symptomatic	−1.249 ± 2.157	0.213
Shoot/root ratio	**Intercept**	**26.912 ± 0.065**	**<0.001**
	Variety	−0.244 ± 0.069	0.807
	**Uninfected**	**6.540 ± 0.061**	**<0.001**
	Symptomatic	−1.220 ± 0.073	0.224
	**Variety-Uninfected**	**−4.084 ± 0.087**	**<0.001**
	Variety-Symptomatic	1.840 ± 0.099	0.067
Dry weight (g)	**Intercept**	**19.884 ± 0.918**	**<0.001**
	**Variety**	**−5.174 ± 0.771**	**<0.001**
	Uninfected	−1.703 ± 0.680	0.090
	Symptomatic	−1.333 ± 0.816	0.184
	Variety-Uninfected	1.532 ± 0.966	0.127
	Variety-Symptomatic	0.717 ± 1.104	0.474
Plant height (mm)	**Intercept**	**12.322 ± 11.418**	**<0.001**
	**Variety**	**−11.414 ± 4.449**	**<0.001**
	**Uninfected**	**5.927 ± 3.928**	**<0.001**
	Symptomatic	1.032 ± 4.717	0.303
	**Variety-Uninfected**	**−3.337 ± 5.573**	**0.001**
	**Variety-Symptomatic**	**−2.164 ± 6.373**	**0.031**

atLEAF value represent the amount of chlorophyll content in the leaves of lettuce.

**Table 2:** Effect of the plant variety and *B. cinerea* infection status on mean (±S.E.) lettuce traits in the sentinel experiment. Abbreviations—U: uninfected; A: asymptomatic infected; S: symptomatic infected; LG: Little Gem variety; TT: Tom Thumb variety.

Treatment	n	atLEAF Value	Plant Height (mm)	Dry Weight (g)	Shoot/Root Ratio
ULG	42	34.511 ± 0.722 _a_	161.683 ± 3.000 _a_	17.254 ± 0.691 _abc_	2.145 ± 0.073 _a_
ALG	25	26.893 ± 0.794 _bcd_	135.021 ± 5.403 _bc_	18.639 ± 0.532 _abc_	1.735 ± 0.033 _bcdef_
SLG	20	27.826 ± 2.071 _bcde_	151.038 ± 3.959 _bc_	16.810 ± 0.505 _abc_	1.688 ± 0.030 _bcdef_
UTT	44	24.936 ± 0.821 _bcde_	93.100 ± 2.420 _de_	14.698 ± 0.253 _def_	1.779 ± 0.010 _bcdef_
ATT	22	23.156 ± 1.399 _cde_	86.937 ± 3.100 _def_	14.467 ± 0.423 _def_	1.724 ± 0.017 _bcdef_
STT	26	19.372 ± 1.452 _f_	82.661 ± 2.985 _ef_	13.861 ± 0.417 _def_	1.831 ± 0.048 _bcdef_

Differences among treatment were examined by means of post hoc Tukey tests (*p* < 0.05). Means within columns followed by the same letters are not significantly different.

**Table 3:** Counts of insect species found on the plants in the sentinel experiment. Abbreviations—U: uninfected; A: asymptomatic infected; S: symptomatic infected; LG: Little Gem variety; TT: Tom Thumb variety. Number of replicate plants shown below each treatment heading.

Species	ULG (42)	ALG (25)	SLG (20)	UTT (44)	ATT (22)	STT (26)	Total
Aphids	Numbers of insects						
*Myzus persicae*	319	1252	81	1847	403	249	4151
*Myzus ornatus*	28	0	0	371	12	3	414
*Macrosiphum euphorbiae*	2173	1331	195	4469	818	204	9190
*Acyrthosiphon lactucae*	2702	1255	232	4737	2295	873	12,094
*Aphis fabae*	40	0	0	409	96	61	606
Total	5262	3838	508	11,833	3624	1390	26,455
Parasitoids							
*Asaphes vulgaris*	6	2	0	28	3	0	39
*Alloxysta victrix*	19	2	0	41	3	1	66
*Dendrocerus carpenteri*	7	2	0	14	3	0	26
*Aphidius ervi*	37	12	1	75	8	3	136
*Diaeretiella rapae*	21	10	2	24	3	0	60
*Praon gallicum*	18	4	0	22	2	2	48
*Aphidius matricariae*	7	5	0	21	5	1	39
Total	115	37	3	225	27	7	414
**Predators**							
*Harmonia axyridis*	4	1	0	2	2	1	10
*Propylea quattuordecimpunctata*	2	1	0	9	6	0	18
*Coccinella septempunctata*	8	0	0	13	1	1	23
*Episyrphus balteatus*	5	1	0	7	0	0	13
*Syrphus ribesii*	4	1	0	9	6	0	20
*Anthocoris* sp.	2	3	0	6	0	1	12
*Tachyporus* sp.	1	0	1	5	0	0	7
Lacewing (Family: Chrysopidae)	0	4	0	5	0	0	9
Total	26	11	1	56	15	3	112

**Table 4:** Summary of the effects of plant variety and *B. cinerea* infection status on the cumulative number of aphids, parasitoid mummies and predatory insects in the sentinel experiment. Significant values are in bold.

Insects	Explanatory Variable	Coefficient z Value ± SE	*p*
Aphids	**Intercept**	**3.171 ± 1.161**	**0.001**
	Host plant variety	0.893 ± 0.133	0.371
	Uninfected	−0.162 ± 0.119	0.873
	**Symptomatic**	**−3.141 ± 0.166**	**0.001**
	**Variety-Uninfected**	**4.267 ± 0.168**	**<0.001**
	Variety-Symptomatic	−0.981 ± 0.222	0.325
Parasitoid mummies	Intercept	−1.487 ± 0.375	0.137
	Host plant variety	1.057 ± 0.295	0.290
	**Uninfected**	**4.079 ± 0.204**	**<0.001**
	**Symptomatic**	**−3.162 ± 0.375**	**0.001**
	**Aphid**	**2.869 ± 0.001**	**0.004**
	Variety-Aphid	−1.474 ± 0.001	0.140
Predators	**Intercept**	**−2.152 ± 0.905**	**0.031**
	**Host plant variety**	**2.712 ± 0.277**	**0.006**
	**Uninfected**	**2.904 ± 0.288**	**0.003**
	Symptomatic	−1.900 ± 0.568	0.057
	Aphid	−0.330 ± 0.001	0.741

**Table 5:** Summary of the effects of plant variety and *B. cinerea* infection status on plant traits following analysis in the established aphid colony experiment. Significant values are in bold.

Plant Traits	Explanatory Variable	Coefficient t Value ± SE	*p*
Chlorophyll index	**Intercept**	**50.392 ± 0.045**	**<0.001**
	**Variety**	**−16.721 ± 0.053**	**<0.001**
	**Plant exposure**	**−2.641 ± 0.050**	**0.009**
	**Uninfected**	**12.063 ± 0.048**	**<0.001**
	**Symptomatic**	**−3.018 ± 0.052**	**0.003**
	Variety–Plant exposure	1.469 ± 0.074	0.144
Shoot/root ratio	Intercept	0.374 ± 0.046	0.708
	**Variety**	**−2.500 ± 0.090**	**0.013**
	Plant exposure	−6.663 ± 0.048	0.508
	Uninfected	−0.539 ± 0.054	0.590
	Symptomatic	−0.496 ± 0.069	0.620
	**Variety–Plant exposure**	**2.951 ± 0.070**	**0.003**
	Variety–Uninfected	1.075 ± 0.099	0.284
	Variety–Symptomatic	1.144± 0.104	0.254
Dry weight (g)	**Intercept**	**74.816 ± 0.062**	**<0.001**
	**Variety**	**−7.176 ± 0.119**	**<0.001**
	Plant exposure	−0.802 ± 0.063	0.424
	Uninfected	1.195 ± 0.072	0.243
	**Symptomatic**	**−2.00 ± 0.092**	**0.046**
	**Variety–Plant exposure**	**2.994 ± 0.093**	**0.003**
	Variety–Uninfected	−0.147 ± 0.131	0.883
	Variety–Symptomatic	1.548 ± 0.139	0.123
Plant height (mm)	**Intercept**	**43.112 ± 0.202**	**<0.001**
	**Variety**	**−6.162 ± 0.389**	**<0.001**
	**Plant exposure**	**−2.807 ± 0.208**	**0.005**
	Uninfected	0.234 ± 0.236	0.815
	Symptomatic	0.313 ± 0.301	0.754
	**Variety–Plant exposure**	**2.195 ± 0.304**	**0.029**
	Variety–Uninfected	0.598 ± 0.429	0.551
	Variety–Symptomatic	−0.082 ± 0.453	0.935

**Table 6:** Effect of the plant variety and *B. cinerea* infection status on mean (±S.E.) lettuce traits in the established colony experiment. Abbreviations—U: uninfected; A: asymptomatic infected; S: symptomatic infected; LG: Little Gem variety; TT: Tom Thumb variety.

Treatment	Chlorophyll Index	Plant Growth (mm)	Dry Weight (g)	Shoot/Root Ratio
Protected from aphid natural enemies				
ULG	8.841 ± 0.311 ^ag^	77.657 ± 3.912 ^abcghi^	23.200 ± 0.540 ^abcghi^	1.110 ± 0.066 ^abdef^
ALG	4.982 ± 0.233 ^bh^	77.525 ± 3.792 ^abcghi^	20.591 ± 0.696 ^abcghi^	0.964 ± 0.053 ^abdef^
SLG	4.390 ± 0.169 ^bcdhij^	76.832 ± 4.197 ^cghi^	19.431 ± 0.984 ^cghl^	0.921 ± 0.088 ^abdf^
UTT	4.330 ± 0.099 ^cdhij^	47.551 ± 2.981 ^defjkl^	14.736 ± 0.544 ^dikl^	0.873 ± 0.033 ^abde^
ATT	1.877 ± 0.130 ^efkl^	32.810 ± 1.719 ^defjkl^	15.076 ± 1.541 ^defjl^	0.787 ± 0.060 ^abdef^
STT	1.499 ± 0.089 ^efkl^	40.440 ± 1.725 ^defjkl^	14.583 ± 0.306 ^efjk^	0.900 ± 0.020 ^abdef^
Exposed to aphid natural enemies				
ULG	7.113 ± 0.443 ^ag^	68.823 ± 4.653 ^abcghi^	21.341 ± 0.753 ^abcghi^	0.900 ± 0.053 ^abdef^
ALG	5.063 ± 0.667 ^bcdhij^	65.955 ± 4.920 ^abc6ghi^	22.042 ± 0.613 ^abchghi^	1.111 ± 0.075 ^abdef^
SLG	4.847 ± 0.211 ^cdhij^	69.396 ± 4.800 ^abcghi^	20.023 ± 1.182 ^abdghijk^	1.051 ± 0.088 ^bcef^
UTT	3.711 ± 0.172 ^ij^	40.950 ± 1.745 ^defjkl^	17.021 ± 1.091 ^efijkl^	1.096 ± 0.093 ^adef^
ATT	2.165 ± 0.263 ^cdefhjkl^	46.680 ± 7.177 ^defjkl^	15.788 ± 0.723 ^dfijkl^	1.000 ± 0.080 ^abdef^
STT	1.539 ± 0.100 ^efkl^	43.167 ± 2.183 ^defjkl^	16.385 ± 0.447 ^edejkl^	1.058 ± 0.041 ^abdef^

Differences among treatments were examined by post hoc Tukey tests (*p* < 0.05). Means within columns followed by the same letters are not significantly different.

**Table 7:** Summary of the effects of the plant variety and *B. cinerea* infection status on the number of aphids per recording day in the established aphid colony experiment. Significant values are in bold.

Explanatory Variable	numDF	F-Value	*p*
**Intercept**	**1**	**410.723**	**<0.001**
**Plant exposure**	**1**	**7.335**	**0.006**
**Plant status**	**2**	**4.774**	**0.008**
**Plant variety**	**1**	**53.584**	**<0.001**
**Day**	**10**	**107.291**	**<0.001**
Plant exposure–Plant status	2	2.749	0.064
**Plant exposure–Plant variety**	**1**	**13.820**	**0.001**
**Plant status–Plant variety**	**2**	**21.739**	**<0.001**
**Plant exposure–Day**	**10**	**85.900**	**<0.001**
Plant status–Day	20	1.384	0.119
**Plant variety–Day**	**10**	**13.433**	**<0.001**
Plant exposure–Plant status–Plant variety	2	1.236	0.290
Plant exposure–Plant status–Day	20	1.079	0.364
**Plant exposure–Plant variety–Day**	**10**	**15.465**	**<0.001**
**Plant status–Plant variety–Day**	**20**	**1.830**	**0.0139**
**Plant exposure–Plant status–Plant variety–Day**	**20**	**1.806**	**0.0157**

**Table 8:** Counts of aphid natural enemies found on experimental plants in the established colony experiment. Abbreviations—U: uninfected; A: asymptomatic infected; S: symptomatic infected; LG: Little Gem variety; TT: Tom Thumb variety. Number of replicate plants as shown below each treatment heading.

Species	ULG (20)	ALG (10)	SLG (7)	UTT (9)	ATT (6)	STT (14)	Total
Parasitoids	Number of insects						
*Asaphes vulgaris*	17	0	0	12	0	4	33
*Aphidius ervi*	27	5	6	39	9	23	109
*Diaeretiella rapae*	27	1	3	6	0	12	49
*Praon gallicum*	32	21	17	3	7	21	101
*Aphidius matricariae*	45	7	14	16	3	17	102
Total	148	34	40	76	19	77	394
Predators							
*Harmonia axyridis*	35	15	16	20	3	6	95
*Coccinella septempunctata*	42	5	4	4	8	16	79
*Adalia bipunctata*	34	1	2	19	4	15	75
*Episyrphus balteatus*	17	5	2	16	1	5	46
*Syrphus ribesii*	12	0	1	5	0	2	20
Total	140	26	25	64	16	44	315

**Table 9:** Summary of the effects of plant variety and *B. cinerea* infection status on the cumulative number of parasitoid mummies and predatory insects. Significant values are in bold.

	Explanatory Variable	Coefficient z Value ± SE	*p*
Parasitoid mummies	**Intercept**	**4.090 ± 0.165**	**<0.001**
	**Variety**	**−2.596 ± 0.104**	**0.009**
	**Uninfected**	**3.440 ± 0.137**	**<0.001**
	**Symptomatic**	**3.825 ± 0.141**	**<0.001**
	**Aphid**	**7.465 ± 0.001**	**<0.001**
Predatory insects	**Intercept**	**3.911 ± 0.217**	**<0.001**
	Variety	−0.231 ± 0.134	0.817
	**Uninfected**	**5.594 ± 0.173**	**<0.001**
	Symptomatic	1.097 ± 0.198	0.273
	Aphid	0.748 ± 0.001	0.455

## Data Availability

All data are provided in the manuscript.

## Supplementary Materials

The following supporting information can be downloaded at: https://www.mdpi.com/article/10.3390/insects15050347/s1, Table S1: Network level statistics for Tom Thumb in the sentinel experiment, comparing observed values to the 95% confidence limits from the null model and including the standardised effect size (SES); Table S2: Network-level statistics for Little Gem in the sentinel experiment, comparing observed values to the 95% confidence limits from the null model and including the standardised effect size (SES); Table S3: Network-level statistics for Tom Thumb plants in the established aphid colony experiment, comparing observed values to the 95% confidence limits from the null model and including the standardised effect size (SES); Table S4: Network-level statistics for Little Gem plants in the established aphid colony experiment, comparing observed values to the 95% confidence limits from the null model and including the standardised effect size (SES).
