# Supplementary material for: Does This Look Infected? Hidden Host Plant Infection by the Pathogen Botrytis cinerea Alters Interactions between Plants, Aphids and Their Natural Enemies in the Field
Source: Insects. 2024 May 12;15(5):347. doi: 10.3390/insects15050347 (PMC11121772; doi:10.3390/insects15050347)
Supplement: Supplementary file 1 [file insects-15-00347-s001.zip › insects-2965110-supplementary.pdf]

SUPPLEMENTARY

Supplementary table S1: Network level statistics for Tom Thumb in the sentinel experiment, comparing observed values to the 95% confidence limits from the null model and including the standardised effect size (SES).

Insect guild	Metric	observed	Null	Lower.CL	Upper.CL	Test	SES
Aphids	Weighted nestedness	-0.643	-0.373	-0.432	-0.307	Lower	-7.248
	Linkage density	2.706	3.040	3.035	3.044	Lower	-103.743
	Weighted connectance	0.338	0.380	0.379	0.380	Lower	-103.743
	Interaction evenness	0.723	0.817	0.816	0.819	Higher	-114.169
Parasitoids	Weighted nestedness	-0.005	-0.834	-0.950	-0.735	Higher	11.991
	Linkage density	3.772	4.330	4.258	4.372	Lower	-15.171
	Weighted connectance	0.377	0.433	0.425	0.437	Lower	-15.171
	Interaction evenness	0.775	0.922	0.910	0.929	Lower	-23.15
Predators	Weighted nestedness	0.736	0.300	-0.071	0.621	Higher	1.886
	Linkage density	3.966	4.398	4.210	4.592	Lower	-3.577
	Weighted connectance	0.360	0.399	0.382	0.417	Lower	-3.577
	Interaction evenness	0.902	0.930	0.914	0.945	Lower	-2.368

Supplementary table S2: Network-level statistics for Little Gem in the sentinel experiment, comparing observed values to the 95% confidence limits from the null model and including the standardised effect size (SES).

**Insect guild**	**Metric**	**observed**	**Null**	**Lower.CL**	**Upper.CL**	**Test**	**SES**
Aphids	Weighted nestedness	0.323	-0.310	0.428	0.189	Higher	2.775
	Linkage density	2.468	2.742	2.734	2.747	Lower	-57.652
	Weighted connectance	0.308	0.342	0.341	0.3434	Lower	-57.652
	Interaction evenness	0.773	0.745	0.741	0.758	Higher	4.638
Parasitoids	Weighted nestedness	0.317	-0.189	-0.644	0.295	Higher	1.254
	Linkage density	3.751	4.093	3.931	4.166	Lower	-4.417
	Weighted connectance	0.375	0.409	0.393	0.416	Lower	-4.417
	Interaction evenness	0.852	0.893	0.871	0.908	Lower	-3.58
Predators	Weighted nestedness	0.414	0.458	-0.231	0.846	ns	-0.126
	Linkage density	3.500	4.206	3.799	4.556	Lower	-2.736
	Weighted connectance	0.318	0.382	0.345	0.414	Lower	-2.736
	Interaction evenness	0.905	0.950	0.931	0.963	Lower	-4.179

Supplementary Table S3: Network-level statistics for Tom Thumb plants in the established aphid colony experiment, comparing observed values to the 95% confidence limits from the null model and including the standardised effect size (SES).

Insect guild	Metric	observed	Null	Lower.CL	Upper.CL	Test	SES
Parasitoids	Weighted nestedness	0.472	-0.56	-0.356	0.154	Higher	3.266
	Linkage density	3.136	3.465	3.337	3.555	Lower	-4.547
	Weighted connectance	0.392	0.433	0.417	0.444	Lower	-4.547
	Interaction evenness	0.902	0.908	0.877	0.927	ns	-0.403
Predators	Weighted nestedness	0.161	-0.010	-0.385	0.213	ns	0.803
	Linkage density	3.237	3.622	3.518	3.714	Lower	-5.275
	Weighted connectance	0.404	0.452	0.439	0.464	Lower	-5.275
	Interaction evenness	0.901	0.938	0.918	0.956	Lower	-2.830

Supplementary Table S4: Network-level statistics for Little Gem plants in the established aphid colony experiment, comparing observed values to the 95% confidence limits from the null model and including the standardised effect size (SES).

Insect guild	Metric	observed	Null	Lower.CL	Upper.CL	Test	SES
Parasitoids	Weighted nestedness	0.853	-0.382	-0.636	0.226	Higher	4.316
	Linkage density	3.238	3.525	3.438	3.589	Lower	-5.456
	Weighted connectance	0.404	0.440	0.429	0.448	Lower	-5.456
	Interaction evenness	0.889	0.914	0.888	0.936	ns	-1.612
Predators	Weighted nestedness	0.165	-0.634	-0.841	-0.465	Higher	7.020
	Linkage density	3.105	3.475	3.362	3.568	Lower	-5.368
	Weighted connectance	0.388	0.434	0.420	0.446	Lower	-5.368
	Interaction evenness	0.822	0.903	0.870	0.926	Lower	-4.547
